# Teleradiology and malpractice: interpreting the risk in context

**DOI:** 10.1186/s13244-025-02032-3

**Published:** 2026-06-03

**Authors:** Mustafa S. Alhasan, Ahmed Y. Azzam, Anjali Agrawal, Arjun Kalyanpur

**Affiliations:** 1 Consultant Teleradiologist, Teleradiology Solutions, Ardmore, PA USA; 2 Assistant Professor of Radiology and Consultant Radiologist, Department of Internal Medicine, College of Medicine, Taibah University, Madinah, Saudi Arabia; 3 Director of Clinical Research and Clinical Artificial Intelligence, ASIDE Healthcare, Lewes, DE USA

Teleradiology has become a cornerstone of modern imaging practice, enabling round-the-clock radiologic services and helping address workforce shortages. However, recent discussions in *Radiology* have raised concerns about its safety profile. Schaffer et al analyzed U.S. malpractice claims data from 2010 to 2022, reporting that teleradiology claims were more likely to involve patient death, cerebrovascular diagnoses, communication failures, and larger indemnity payouts compared to non-teleradiology claims [1]. Mezrich’s accompanying editorial suggested that the nature of remote interpretation itself might elevate malpractice risk [2]. Yet, such conclusions may oversimplify a complex dynamic of clinical, operational, and structural variables.

A central issue with the analysis by Schaffer et al is the lack of case matching. The teleradiology and non-teleradiology cohorts were not adjusted for exam type, time of day, acuity, or subspecialty. Teleradiology comprised only 4% of total claims, yet these cases were disproportionately severe. For instance, 35.6% of them involved death, compared to 19.7% in traditional claims [1]. Without adjusting for patient and exam characteristics, comparing these datasets risks attributing outcome disparities to practice setting rather than case context.

Most teleradiology services are designed to provide after-hours emergency coverage. This traditional model frequently handles high-risk, high-acuity cases such as trauma or stroke, typically interpreted at night when clinical communication is more fragmented. For example, cerebrovascular diagnoses were nearly three times more frequent in teleradiology claims than in traditional ones, reported at 9.6% vs 3.6%, respectively [1]. These figures are more likely to reflect patient acuity rather than the limitations of remote interpretation.

Circadian misalignment and fatigue add to these challenges. A Mayo Clinic study showed that overnight shifts are associated with a higher rate of radiologic errors, with the rate rising to 3.7% in the early morning hours [3]. When teleradiologists interpret complex studies in the middle of the night without full clinical context, their diagnostic burden increases considerably. These working conditions, rather than geographic distance, may better explain the observed rise in claim severity.

Teleradiologists also often face intense productivity pressures. A $15.5 million malpractice verdict recently highlighted a case in which a teleradiologist read two CT scans in 5 min, missing a cervical spine fracture that resulted in quadriplegia [4]. The accelerated pace of interpretation was central to the negligence claim. In many systems, high volume and rapid turnaround expectations are incentivized by compensation structures that emphasize speed. This environment, while efficient, may compromise diagnostic caution.

The relationship between workload and error is well established. A review in *Radiology* emphasized that increasing reading speed and prolonged shifts undermine diagnostic accuracy [5]. Similarly, a large retrospective analysis of 2.9 million exams found decreased accuracy during overnight shifts and under high-volume conditions [6]. These findings are not exclusive to teleradiology, but the effects are amplified in remote practice due to logistical and communication barriers.

Sub-specialization is another important factor. While many teleradiologists are experienced generalists, they may read across multiple domains, such as neuro, body, and musculoskeletal imaging in a single shift. In contrast, hospital-based radiologists often focus on a single area of sub-specialty, improving sensitivity for subtle findings. Emergency radiology has evolved into a recognized subspecialty for a reason. Its complexity warrants focused expertise. Routing cases to subspecialists, even in remote settings, may enhance accuracy and reduce risk.

Communication challenges frequently contribute to malpractice claims. Teleradiologists typically work without direct access to clinicians or complete clinical records. This lack of real-time context can result in delays or misinterpretation. Schaffer et al noted communication failures in 25.9% of teleradiology claims, compared to 12.6% in conventional claims [1]. Communication errors are a known contributor to malpractice risk in radiology [7]. The core issue here is not physical distance, but an underdeveloped communication infrastructure, especially given that teleradiology practices often provide coverage for over 100 hospitals, each with their own communication protocols and platforms.

Digital transformation has helped narrow the gap between remote and on-site workflows. However, communication systems have not always kept pace. On-site radiologists can quickly clarify findings in person, while teleradiologists must rely on phone calls or messaging platforms that may be asynchronous or poorly integrated. The solution lies in investing in robust tools and protocols such as standardized alerts, embedded chat systems, and virtual multidisciplinary meetings.

Teleradiology is not inherently more dangerous. Its increased exposure to complex and urgent cases, combined with limited communication and time constraints, exacerbates risk in certain situations. When contextual variables are accounted for, several studies suggest that diagnostic accuracy in teleradiology is comparable to in-house radiology [8]. A systematic review even demonstrated low discrepancy rates across international teleradiology services, particularly when quality assurance processes were in place [9].

Recent malpractice analyses should not erode confidence in teleradiology. Instead, they should prompt efforts to improve the systems in which remote radiologists operate. Encouraging matched cohort studies, refining incentive structures, integrating AI-driven triage, and strengthening communication protocols are all meaningful ways forward.

Teleradiology represents an essential evolution in radiologic care. It expands access, ensures timely diagnosis, alleviates radiologist shortages in remote or underserved areas, and supports healthcare systems facing growing demands. To ensure its continued success, the focus should shift away from questioning the location of radiologists and instead prioritize optimizing the environment in which they work.

## Data Availability

Not applicable.
